# Assessment of multiple subjects' synergetic governance in vocational education

**DOI:** 10.3389/fpsyg.2022.947665

**Published:** 2022-09-13

**Authors:** Min Wu, Md Nazirul Islam Sarker

**Affiliations:** ^1^School of Public Administration, Sichuan University, Chengdu, China; ^2^School of Social Sciences, Universiti Sains Malaysia, Pulau Pinang, Malaysia

**Keywords:** educational psychology, new education paradigm, educational governance, teaching-learning, synergetic approach, multi-subjects' approach, organizational psychology

## Abstract

Synergetic governance is a practical approach to ensure quality in the teaching-learning process at multi-dimensional perspectives. This study intends to explore the potential of a synergetic governance approach in the vocational education system. A systematic literature review has been done by applying the PRISMA approach. The last 21 years' literature has been analyzed, and a synergetic governance model has been developed. This study reveals that the synergetic governance of education deals with integrating all available resources to enhance development by meeting the demands of market, society, and nation as a new paradigm. An effective vocational education system requires the participation of all related stakeholders in the governance system. This study has developed an “intervention-approach-synergy-integration” mechanism to show how the education elements are connected to each other. The study reveals that the major synergetic elements are openness, equilibrium, dynamism and cooperation. To ensure synergetic governance, these elements should be integrated with an educational institution, market, public, society, state and private organization. It argues that the synergetic governance of these related components can reduce the demand and supply gap of quality vocational graduates in the industry – academy sectors. This study recommends incorporating multi-subjects' synergetic governance to expedite the marketable quality of vocational education.

## Introduction

The synergetic approach is a meaningful way to convey critical information holistically rather than focusing on a single point. Solving complexity in a teaching-learning system that focuses on multi-dimensional perspectives is a cognizable process. The synergetic approach deals with the complexities of modern education to make it simple to produce quality graduates (Panina et al., 2017). This approach utilizes all the available resources to explore a learner's potential and creative personality (Dvoenosova, 2016). Nowadays, pedagogical education has a great demand due to the change in socio-cultural phenomena. A synergetic approach can be a key pedagogical educationmethod to nurture a learner's intellectual potential. It can produce future specialists by recognizing students' self-worth, creative thinking, and solving inconsistencies in the teaching-learning process (Aldaihani, 2017). It stimulates self-organization to solve all complexities related to the heterogenous system and non-equilibrium structures and promotes the self-actualization of the learner (Mao and Wang, 2018).

Traditional vocational education focuses on one-way learning and training facilities incompatible with market demand (Mouzakitis, 2010). There is always a gap between the supply in the vocational field. One way focuses on a narrow perspective of learning and fails to meet the demand of the market, society, and nation. Only a multi-dimensional, multi-subject, and holistic way of teaching-learning process can produce quality graduates and personnel for future development (Gao, 2018). This multi-dimensional and intellectual graduate can be produced using a synergetic approach (Ting-lin and Jing, 2019). The value-oriented, multi-dimensional and integrated teaching-learning process can be ensured by a synergetic approach to pedagogical system development through openness, co-creation, and value orientation in education (Liu et al., 2018).

Modern governance is always dealt with authority and responsibility. Education quality management depends on quality-centered governance comprising a quality relationship between rights and responsibilities (Keep, 2015). It helps to achieve the goal of government through a necessary combination between various rights and duties. The main three factors of education quality management are state, educational institution, and society (Gore and Morrison, 2001). Synergetic governance provides an opportunity to deal holistically with the state, educational institutions, and society to provide quality graduates (Lu et al., 2017). Multiple subjects' synergetic governance helps to produce quality graduates through making an interdisciplinary link between various subjects and prioritizing the demand of the market, society and state (Mao and Wang, 2018). For ensuring educational quality, a synergetic governance approach requires a continuous process of quality improvement actions through institutional arrangement and ongoing governance following the primary quality objectives and standards. Synergetic governance involves the common function and game of multi-party governance subjects for coordinating the relationship among the governance rights of all parties (Hill et al., 2016). This process can form a consistent joint force in synergetic governance for improving the quality of vocational education.

Synergetic governance for vocational education depends on integrating the market demand, standards, target, and development nature. It provides an opportunity to make harmonious symbiosis among various elements of the governing process. The synergetic governance is better than the traditional one-way education system because it properly emphasizes all related aspects and integration (Mao and Wang, 2018).

Suppose the obligations of colleges and universities in independent quality management mainly come from laws and regulations. In that case, the responsibility of their autonomous quality management is the corresponding “in-part responsibility” that they enjoy the power (Martasari et al., 2018). This kind of “sub-responsibility” mainly includes the educational responsibility that colleges and universities should bear as the main body of higher education implementation and social responsibility as the essential components of the social subject (Chen and Liu, 2016). On the one hand, colleges and universities are the specialized agencies and the main body of higher education, and their fundamental activity is education and teaching. Ensuring the quality of education and teaching is the legal obligation of colleges and universities and the first responsibility it should fulfill (Gibb, 2012). Therefore, for colleges and universities, self-quality supervision, improvement, evaluation, and constant improvement of the internal quality management system and structure, will continue to improve the quality of education and teaching. As the core work of internal quality management, personnel training quality is the university to achieve internal quality improvement should bear the educational responsibility (Ting-lin and Jing, 2019). Universities need to draw extensively on social opinions, absorb social advantages, and actively explore an internal and external quality governance integration mechanism or model suitable for developing colleges and universities (Thoenig and Paradeise, 2014). Colleges and universities better serve society and economic development by optimizing the quality of higher education to improve the external quality benefits. This is also the social responsibility of colleges and universities to achieve exterior quality improvement (Chen and Liu, 2016).

Literature shows that several studies have been done on public education service (Mao and Wang, 2018), community education (Swan et al., 2002), teaching-learning process (Peña-López, 2016), and synergetic approach in public services (Zeng et al., 2007; Sierens et al., 2009; Aldaihani, 2017; Panina et al., 2017); but still lack synergetic governance on vocational education system. As a holistic approach, the synergetic governance approach can be a potential tool for producing quality marketable vocational graduates in developing and developed countries. Therefore, this study explores the potential of a synergetic governance approach in the vocational education system. The remaining section of this article presents the methodology in Section Methodology, results in Section Results, discussion in Section Discussion, and conclusion in its last Section.

## Methodology

### Research design

A comprehensive literature review has been conducted over the last 21 years. Information about the most recent innovations has been compiled to contribute to the discussion on the teaching-learning process. This study highlights a new governance paradigm in education that can play a vital role in producing quality vocational graduates.

### Search strategy

Literature reviews can help to establish a new topic of study and research. It usually provides an opportunity to review, integrate, and focus prior studies on uncovering fresh information that can aid in developing a new teaching and research paradigm. Keeping these philosophies in mind, this study extensively searched several renowned databases like the web of science, engineering village, Scopus, and google scholar using relevant keywords such as synergetic, vocational education, educational paradigm, teaching-learning process, and educational governance. This systematic literature review was done from July to August 2021.

### Inclusion and exclusion criteria

Two inclusion criteria have been followed to select quality documents: (a) Does this study focus on educational governance and the teaching-learning process? (b) Does it deal with the synergetic approach or any related approach for producing quality graduates? This study also considers the related studies based on primary data and systematic literature reviews to present solid arguments for synergetic governance education as a new paradigm.

## Results

### Systematic review results

**Figure 1:**
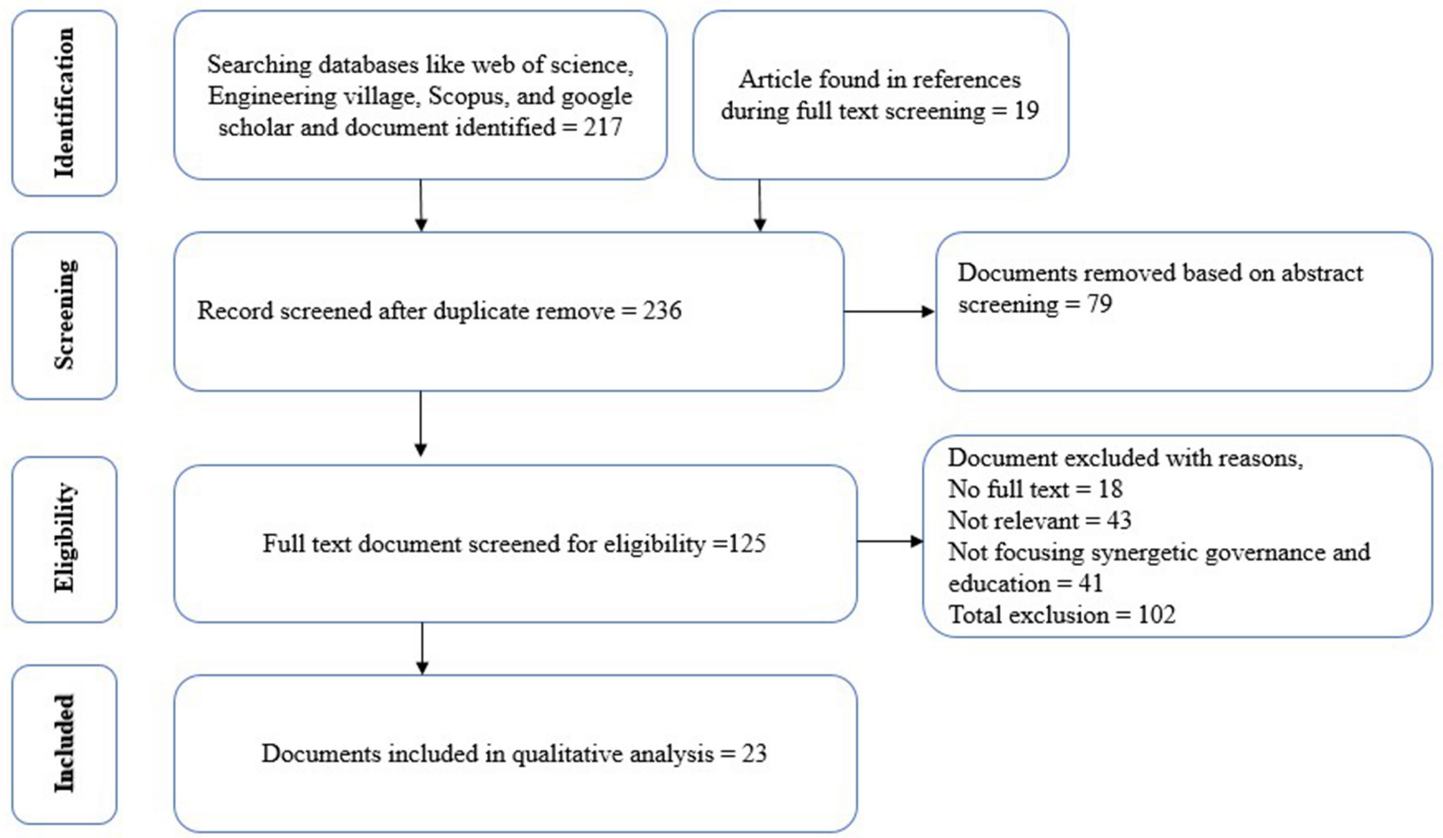
Document selection by PRISMA analysis.

This study is largely directed by systematic review and meta-analysis (PRISMA) checklists. The major phases of PRISMA checklists are identification, screening, eligibility, and inclusion. In the identification stage, 217 quality documents were selected along with 19 other references. In the screening stage, 79 documents have been removed after careful abstract screening through this study's inclusion and exclusion criteria. In the eligibility stage, 125 quality documents were selected by eliminating 102 papers for several reasons, such as no full text, non-relevancy, and not focusing on synergetic governance and education. In the inclusion stage, 23 quality documents have been selected comprised of journal articles, book chapters, books, and working papers to explain the potential of synergetic governance for producing quality marketable graduates (Figure 1).

### Analytical results

#### Areas of intervention

The areas of intervention focus on the critical areas of the teaching-learning process, which is very potential for the intervention of synergetic governance. This study has analyzed the relevant literature and identified critical areas of intervention such as student assessment, teacher appraisal, school evaluation, school leader appraisal, and education system evaluation (Table 1). This identification is based on the frequency of the terms, relevancy to the context, and suitability to present the synergy among various components.

**Table 1:** Areas of intervention in the education sector under synergistic governance.

**Areas of intervention**	**Relationship**	**Sources**
Student assessment	Student assessment can ensure the quality of education.	(Thoenig and Paradeise, 2014; Dobbins, 2017)
Teacher appraisal	Teacher appraisal can ensure the quality of teaching and, ultimately, quality graduates.	(Seitz, 2009; Greatbatch and Tate, 2018)
School evaluation	School evaluation can help to improve the education governance that can ensure to produce quality graduates.	(Herrington et al., 2006; Hasanefendic et al., 2016)
School leader appraisal	School leader appraisal can ensure the school's quality, which is very important for vocational education.	(Cheng and Chan, 2015; Wahab and Tyasari, 2020)
Education system evaluation	Education system evaluation can ensure the applicability of the learning and education to the field or industry level.	(Nzonzo, 2017; Jais et al., 2021)

#### Holistic approach

A holistic approach implies looking at something in its entirety and grasping the related components. In the case of the education sector, it comprises governance (Nzonzo, 2017), capacity (Yang, 2018), and implementation (Evans, 1996). Most researchers have emphasized these three approaches. A comprehensive approach is necessary for a balanced and sustainable development of the education ecosystem sectors (Table 2).

**Table 2:** Holistic approach in the education sector under synergistic governance.

**List of approaches**	**Relationship**	**Sources**
Governance	Governance ensures the implementation of the educational policy properly so that educational institutions can produce market-oriented quality graduates. The quality of teaching and learning is largely dependent on design and procedure.	(Souto-Otero, 2013; Lu et al., 2017; Nzonzo, 2017)
Capacity	All level stakeholders should be capable of managing the school properly and ensuring quality education. The teaching and learning approaches should be such which significantly affect the quality of graduates.	(European Training Foundation, 2013; Martasari et al., 2018)
Implementation	The instructional education policy should be implemented properly and in an integrated way.	(Zeng et al., 2007; Rudenko and Morosova, 2015)

#### Synergetic elements

The synergistic interaction mechanism of educational and scientific industry components can provide the opportunity to develop quality graduates with improved characteristics, create an environment for developing policy initiatives, and market the results of quality education (Evans, 1996). This study has analyzed the relevant literature and identified vital elements such as openness, equilibrium, dynamism, and cooperation (Aldaihani, 2017). These elements are related to each other in the education sector (Table 3).

**Table 3:** Synergetic elements in the education sector under synergistic governance.

**List of elements**	**Relationship**	**Sources**
Openness	The educational institution should be open to connecting with the outer world and exporting education-related services.	(Thoenig and Paradeise, 2014; Filho et al., 2018)
Equilibrium	There should be an equilibrium between demand and supply so that industry, society, and the state can benefit from graduates.	(Souto-Otero, 2013; Pouliakas and Ranieri, 2019)
Dynamism	There should be an interaction with each education system and industry stakeholder. The linkage should be dynamic rather than static.	(Mok, 2005; Doh et al., 2021)
Cooperation	Win-win cooperation should be ensured among stakeholders.	(Aldaihani, 2017; Nzonzo, 2017)

#### Integration

Integration focuses on implementing synergistic practices at the educational and industry levels by coordinating institutions, private organizations, society, state, public, and market (Table 4) (Gibb, 2012). Many researchers have emphasized these actors who are active in the synergetic governance practices of the education sector (Herrington et al., 2006; Zeng et al., 2007; Eddington and Eddington, 2011; Gibb, 2012; Superfine et al., 2019).

**Table 4:** Integration in the education sector under synergistic governance.

**List of sectors**	**Relationship**	**Sources**
Educational institution	An educational institution is a key actor in the multiple subjects' synergistic governance.	(Fadeeva and Galkute, 2012; Mao and Wang, 2018)
Private organizations	Private organizations play a vital role in commercializing education and recruiting vocational graduates.	(Lu et al., 2017; Mao and Wang, 2018)
Society	Society should benefit from vocational graduates.	(Gibb, 2012; European Training Foundation, 2013)
State	The state is the largest beneficiary of synergistic education as a policymaker, recruiter, and exporter of quality graduates.	(Evans, 1996; Martasari et al., 2018)
Public	Public interest should be the primary concern of education synergistic governance.	(Hu et al., 2018; Zoellner, 2020)
Market	The market and government relationship should be favorable for ensuring quality vocational education.	(Havemann and Mackinnon, 2002; McGrath, 2012)

## Discussion

This section is presented into several sub-sections; the first sub-section deals with the synergetic governance concept in vocational education, and the second sub-section deals with approaches to synergetic governance in the vocational education and industry sector.

### Synergetic governance concept

The term “Synergetic” is originated from the Greek word that means a science dealing with cooperation and competition among various sub-systems in a system. It also focuses on the transformation rule of a system that is done from disorder to order. Since a system is made by several sub-systems, so transformation rules usually vary from sub-system to sub-system (Yang, 2018). The characteristics, structure, and nature of the various sub-systems are not simple, rather complex. This complexity creates competition within sub-systems in the system's transition process (Superfine et al., 2019). A system can be the best if its sub-systems work correctly through synergetic governance (Doh et al., 2021).

The theory of synergetic education has been developed in the US and applied for educational reform. “Synergy” in the education system can significantly influence producing qualified graduates, that have been proven in developed countries. This approach gained new momentum in education and research with the advancement of technology after 1990. Contemporary researchers have given more attention to the synergy approach in education by supplying open, resourceful, and quality learning resources. Similarly, some researchers also focus on leveraging technology in the educational approach, which helps enhance the synergetic education system. Some individuals and organizations also provide individual and organization-level education.

The concept of synergy education is complex. It deals with training learners for overall development by using talent and contemporary resources and meeting the market, society, and state (Gao, 2018). This approach improves the principles of sharing resources and produces more quality graduates through interaction, strategy, and innovation (Fadeeva and Galkute, 2012). This concept supports ideological and political education, innovation, and enterprise education (Mao and Wang, 2018). Ideological and political education deals with quality education for improving ideology and political insights for solving contradictions and enhancing the development of spiritual civilization (Eddington and Eddington, 2011; Gao, 2018). Innovation and enterprise education deal with two aspects of education: creative ability and entrepreneurship ability for developing quality graduates who can think about innovation and its effective integration into the market (Peña-López, 2016).

### Approaches of synergetic governance

**Figure 2:**
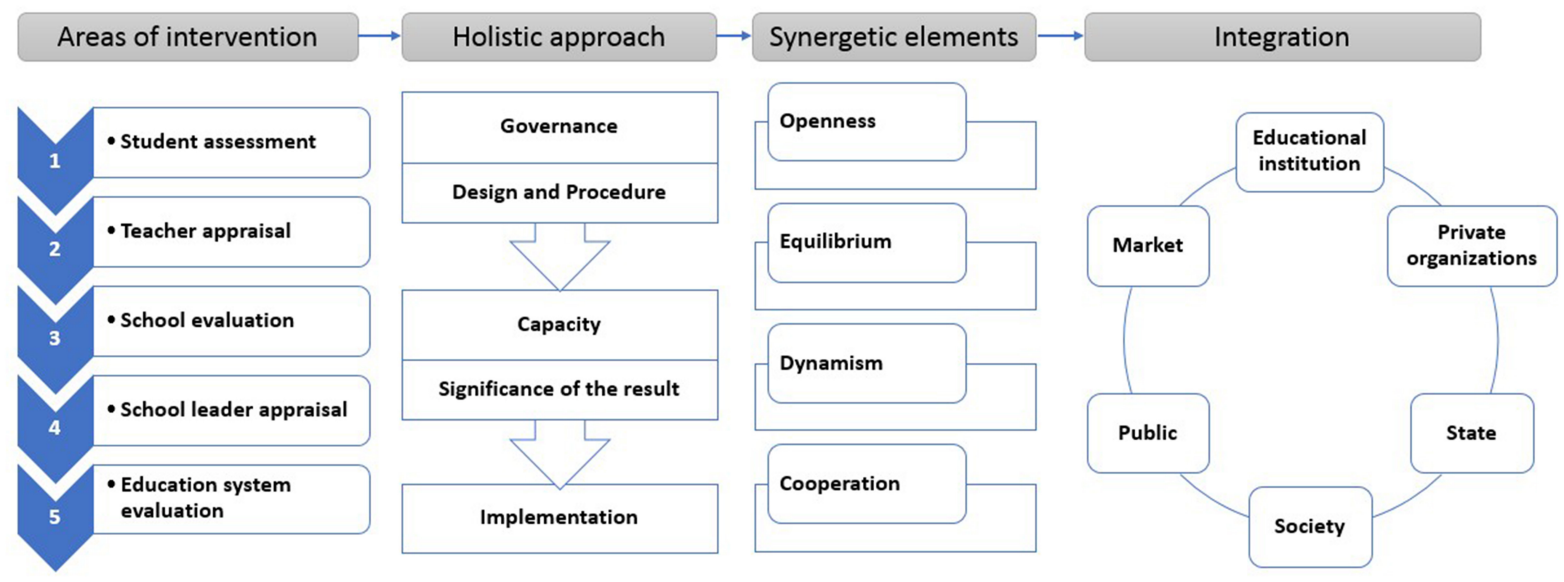
Multiple subjects' synergetic governance in education.

To hush up the contradiction between supply and demand of vocational graduates and achieve the governance goal of vocational achievement, the fundamental action is to promote interdependent governance entities to intensify collective behaviors and form a practical and effective governance mechanism *via* various effective interactions (Mao and Wang, 2018). Based on synergic governance, this study develops the “intervention-approach-synergy-integration” mechanism to show how the various governance elements are connected to each other. The specific model is shown in Figure 2.

#### Areas of intervention

##### Student assessment

The primary assessment methods are initial, formative, and summative assessments. The education institution produces a competency evaluation plan as part of its quality assurance system. The respective education provider can make Competency Assessment Plans for each training or qualifying program (Doh et al., 2021). The project details the guidelines and procedures the training provider has set for carrying out an expert evaluation. The plan covers, among others, how the following aspects are implemented (who, how, where, and how students, staff, and stakeholders are informed of teaching, guidance, and advice staff, as well as expert assessors); prior learning recognition; proof of competence; assurance of abilities before competence demonstration; evaluation, certification. Teachers, guides, and skill assessors apply the competence evaluation plan (Fadeeva and Galkute, 2012). The strategy is monitored and evaluated for its feasibility.

##### Teacher appraisal

The professionalism of teachers is a crucial objective typically used when considering the quality of instructors and teacher education. While professional identity and trainers are necessary for developing skills in the workforce, this function does not provide high prestige (Yang and Ni, 2018). Some two-thirds of workers who form the economic backbone in most industrialized countries are middle-level workers and employees who, through the support of teachers, trainers and trainers in non-academic technical vocation education and human resource development, have learned a substantial portion of work skills and know-how. Vocational teachers can be found in the spectrum of early education in lower secondary schools and adult education centers where adults of various age groups are involved. This is nevertheless just one example: the following differences reflect the complete complexity and structural causes of the work of vocational teachers (Greatbatch and Tate, 2018). The way society organizes the school-to-work transition is a systematic way of characterizing sorts of vocational education systems. In many societies, vocational education and education is simply linked to the conditioning of the non-academic population for specialized jobs rather than a process which almost any member of society undergoes, developing attitudes, skills and knowledge that constitute substantial and necessary resources for the individual to be involved in economic and social life.

##### School evaluation

Monitoring and evaluating the entire supply system are crucial elements like firewalls related to orderly progress and supply objectives. First, the laws and the rules should be developed and improved. The legal framework applicable should be established based on the attributes of primary public education products. The rights, responsibilities, and duties of each supplier entity should be separated so that its supply conduct can be restricted and eliminated arbitrarily. Clarify particular regulation items, such as market access, qualification, quality of service, price monitoring, and public financial inputs, set open and transparent monitoring mechanisms, and execute the relevant administrative reviews and audits (Ting-lin and Jing, 2019). Secondly, the information communication system should be established and improved. The disclosure of the information is a condition for successful supervision and assessment (Thoenig and Paradeise, 2014). If actions are used to prevent and resolve public disputes, they must be fully-informed by the masses.

Consequently, it is important to specify the scope, methods and feedback modalities for each provider to disclose the information; it is the customary notion of openness and non-disclosing as an exception. The relevant information is disseminated to society *via* the principal media to make it public and transparent. Third, it is essential that we take full action concerning the role of the meta-government in diversified supply companies and establish a government-led, diversified multi-dimensional mechanism for monitoring and evaluating the education services as an unassuming task and obligation, and to a certain degree, that can offset market deficiencies. People should be aware to vigorously enhance the function of public opinion supervision to create a 'up and downlinks, internal and external synergy' mechanism for multidimensional supervisory and synergy management (Mao and Wang, 2018). Fourthly, we need to develop and improve the reporting system for performance assessment and the mechanism for accountability in the supply of vocational education and actively implement a third-party assessment system (Zeng et al., 2007). It also needs to publicly publish the performance assessment results to ensure that social supervision is acceptable and quality education services are supported, forming the virtue.

##### Leadership appraisal

Leadership is the driver of change and is well-known at every level. Many institutional leaders reevaluate how to balance their teaching and research missions and improve their teaching and learning quality (Ahmad, 2015). However, top-down approaches may encounter pushback by faculty, which considers that the correct mix of institutional leadership and management interference requires academic freedoms and care impairment (Thoenig and Paradeise, 2014). There has been a lot of improvement despite some resistance. The faculty has been progressively seeking to increase the relevance of its programs to social and economic requirements and is increasingly ready to revisit its role in enhancing student learning and its future employability. Many student support programs explore alternate pedagogies or adapt them to different profiles of students. Policy synergy is aimed at improving teaching and learning. Although money is important, education quality can improve without considerable investments. Prioritization in line with the educational model and the goals defined by the institution will be needed to sustain quality improvements. Not all instructors are innovators, and few innovations without an efficient organizational structure can be distributed and sustained (Herrington et al., 2006). To embrace quality teaching, higher education institutions should be considered learning organizations.

##### Education system evaluation

Literature analysis reveals that the synergistic approach aims to fulfill one of the most complicated goals of the current education system: the transition to creative, problem-based methods of teaching and education that assure the creation and development of a creative personality (Eddington and Eddington, 2011). The synergistic process offers a fresh approach to constructing instructional systems, especially from openness, co-creation, and self-growth orientation (Panina et al., 2017). As higher professional institutions improve, enrollment increases gradually, therefore the number of higher graduates is steadily growing, and their job pressure is increasing. Problems such as creating a better area for graduate development and providing a better platform need immediate resolution in the new era. This study mainly examines effective “synergy” education strategies with education for innovation and business and ideological and political education in college students (Nzonzo, 2017).

One of the main aims of educational universities is to develop staff ready to take management decisions in the setting of uncertainty and upgrade the educational system. Synergy in educational organization is the design of curricula as a picture of development, a change of relationship between topics in the educational process as a path of progressive shifts in the educational environment (Aldaihani, 2017).

#### Holistic approach

Holistic means comprehending discipline-wide interrelations, looking for integrated thoughts and practice (Fadeeva and Galkute, 2012). The study recommends a comprehensive approach to take due to the need for a balanced and sustainable development of the education ecosystem sectors while defining achievable objectives and strategies for strengthening and developing post-secondary education self-finance across an ever more diverse and, to a certain extent, in the competitive environment (Eddington and Eddington, 2011).

The competence governance process often incorporates various education and training, labor markets, and other social players. Creating and building on the labor market and available intelligence skills are referred to as an advancement and a matching information system to establish informed economic development through focused investments in skills by specific countries (Greatbatch and Tate, 2018). Skills governance refers to the process of participation in the generation, transfer, and use of skills and intelligence to implement and steward education and training policies by stakeholders in public, private and third sectors from various economic and geographical industries and units (Pouliakas and Ranieri, 2019). It consists of institutional formal and *ad hoc* bodies, incentive structures, and other methods for steering education and training and guaranteeing the quality of training in line with the intelligence of available talents (Manuel Galvin Arribas, 2016). It includes a negotiating viewpoint considering the education system's short, medium, and long-term needs and the labor market. Appropriate governance arrangements must exist to overcome the challenges and achieve a collaborative approach to ensure that all essential parties are committed to long-term policy and synergistic (Doh et al., 2021).

Management of higher education institutions includes planning, design, coordination, monitoring, assessment, and organization tasks. This material is focused on the organization as a component of the management of educational processes in universities. This study finds the synergetic method as a transfer of integral information blocks that, rather than as a phenomenon or a problem, provide an overall view of the universe, enabling us to focus on the multidimensional, complex, and polyphonic nature of cognizable processes. The socio-cultural transformations in society extend the range of occupations where pedagogical instruction is required (Martasari et al., 2018). The structure and requirements of educational consumers are changing: the field of further vocational training and the proportion of adult education are increasing, and the principle of continuity of education is confirmed.

#### Application of synergetic governance in education

##### Openness

Vocational education services are a complex open system, including demand system, providers, supply strategy, supply mode, supply supervision, and evaluation system (Yang and Ni, 2018). This system requires integrating various social resources, exchanging materials, energies, and information with the outside world and the constant exports of different public education services (Mao and Wang, 2018).

##### Non-equilibrium and equilibrium

Impacted by the dual structure of urban and rural economic and social development, the basic provision of vocational education remains in a non-equilibrium far from the equilibrium in many countries, which presents the supply and demand disequilibrium between the regions, between urban and rural areas, and between schools (Panina et al., 2017). For example, in China, the gross supplies of vocational education in the vast rural areas and former revolutionary base areas are relatively insufficient at a relatively backward level but ample and relatively high level in economically developed areas (Yang and Ni, 2018). In the supply process of vocational education, all supply entities, through interest games, reach consensus by equal dialogue and negotiation in the perspective of integrity, constitute a community of shared interests, follow the rules of the identity game, share risks, and increase supply. It can balance with demands in the synergy governance supply mechanism mode.

##### Dynamics and harmony

Along with developing the economy and society, the public raises increasingly diversified and personalized demands for superior and fair education services based on the changes in social structure (Mao and Wang, 2018). As there are strengths and weaknesses of public needs and the disparities between them, the multiple supply entities in the system present a degree of correlation. It can also ensure the strength of the interaction with each other and the speed of the movement change, all of which dominate the existence of the whole system (Zhu and Peters, 2019). Thus, supplies will increase in total and improve quality and levels. In the supply network structure, each supply entity is more concerned about the dynamic changes in demands, reaching a consensus *via* communication and negotiation, and making full use of in-house resources, knowledge, and technologies to complement each other and optimize the allocation of supply resources and achieve a win-win cooperation (Thoenig and Paradeise, 2014).

##### Cooperation

To ensure a fair and effective supply, communication, negotiation, coordination, and cooperation between the host, the object, and the subsystems are required to operate orderly (Yang, 2018). A win-win partnership based on mutual benefit, respect, and trust indicates a developing trend of vocational education services. Each supply entity maintains a moderate tension and plays a game for power in the macro supply system. Based on the above analysis, the provision of vocational education services fits in with the concept of synergic governance, and the two have greater conformity. For this purpose, we can draw on the theory of synergic governance to study the provision of vocational education services. Under the concept of synergic governance, the provision of vocational education services means a dynamic process where the government, the market, non-governmental organizations, the private sector, and individual citizens constitute an open overall supply system in the development process of basic public education (Aldaihani, 2017). It can promote the optimal allocation of social resources, change the whole supply system from disorder to ordered modes, maximize the maintenance and realization of the public education benefits *via* the synergic governance of BPESs, and finally achieve the objectives as required by public education.

Regarding synergy theory, the synergic provision of vocational education is such a process that government authorities, market, and social organizations communicate and coordinate to co-offer vocational education services (Panina et al., 2017). It may be based on public demands to realize mutual complementarity of advantages, sharing information and resources, under the guidance of certain rules.

#### Integration

Interdisciplinary and intradisciplinary integration is carried out and is a logically finished cross-sectional knowledge framework. This integration adds knowledge to each topic's material, mixes it, and offers an active preparation that develops abilities and personal attributes of a professional nature and not a specific subject (Thoenig and Paradeise, 2014). The interdisciplinary formation of science concepts is facilitated by pedagogical, common teaching, and psychological conditions: a) time-coordinated study of individual academia, each based on a prior conceptual basis, and preparing students to understand the following subject successfully; b) continuity and consistency of the concepts; c) uniformity concerning the interpretation of general scientific concepts; and d) exclusion from the reproduction of the same notions in the analysis of different subjects. These functions allow pupils to have a complete education, hence integrating the educational process. The most proper technique for creating an integrated cognition with the existing subject-block training system is integrated courses. The methods used vary depending on the definition of goals, the degree to which integrated disciplines are integrated into the general problem area, the nature of the interdisciplinary, and, lastly, the creation of a new developer's individuality (Yang and Ni, 2018).

With the existing contradiction between the supply and demand of graduates, we should set up and break through the supply mechanism of education services with a notion of synergistic governance. Essentially, governance is only a technique of coordinating providers with various conceptions and interests to achieve a consensus on collective action (Pouliakas and Ranieri, 2019). The administration is, in fact, a process in which there can be a consensus. To achieve this, social consensus is the cornerstone of innovation in vocational education. The supply entities and their knowledge are all diverse. Suppliers should strengthen their reasoning knowledge to fully understand the importance of vocational education, improve their sense of identity, foster the concept and spirit of synergistic governance and create a suitable social environment for mutual involvement and synergistic governance. Synergistic governance means that every providing company should consider its interests and consider the public interest, integrate actively into society as a social person, participate in general education issues, and share governance responsibility (Lúcio and Neves, 2010).

Synergistic governance focuses on social power involvement and emphasizes the transfer from government to the social organization of public governance functions (Superfine et al., 2019). This means that the graduate must be supplied based on the strength of some social organizations. The NGO is non-governmental, not-for-profit, voluntary, autonomous, and public benefit (Manuel Galvin Arribas, 2016). It has dissolved the dual pattern of resistance between the government and the market, contributing to overcoming government failure and market failure and increasing supply. State power commonly leaves various fields, such as economics, society, and politics, while non-governmental social, civil and intermediary organizations, to fill up the area freed up by State power, take up part of the management of public affairs functions divided from governmental organizations (Evans, 1996). The government ought to promote and develop NGOs energetically. On the one hand, a good institutional framework must be created to operate independently. To protect their legal status, legal rights, and interests first adopt and enhance necessary laws and regulations. Secondly, explain the rights and functions of the Government and NGOs, develop a win-win collaboration, and play the role of “minimum government, great society” governance. The self-building of NGOs, however, is enhanced. First, fully play the management function of its discipline and set up an independent system for its operations. Secondly, staff training is strengthened, and their credentials and skills constantly improved to participate in synergistic public education administration. Finally, to avoid alienation, develop third-party monitoring, evaluation, and accountability procedures for NGOs.

The government assigns rights and interests logically and then makes optimal use of the law, taking suitable steps to execute accurate instructions, if necessary, and eventually establishing a cooperative partnership win-win. To prevent the presence during the providing time and infringe the rights and interest of the masses, of such circumstances that public educational services are not in existence, we must provide public education services, and the state should be responsible for paying guarantees (Panina et al., 2017).

## Conclusion

The synergy approach plays a significant part in the construction of educational management, particularly in producing graduates according to the demand of society, industry, and the nation. The study argues that synergetic education governance aims to integrate all available resources to improve development by responding to market, community, and nation demands as a new paradigm. An effective system of vocational training involves the engagement of all stakeholders involved in the governance process. This study developed a method of 'intervention-approach-synergy-integration' to show how the aspects of education are interconnected. It proposes that this synergetic governance of these associated components could lessen the demand and supply imbalance between quality professional graduates in the industry-academia. Multiple complex system components comprise an integral synergetic system in openness, equilibrium, dynamism and cooperation. Some of these skills are more generic to an educational leader, but others are regarded as obligatory for graduates of any educational school. In the education sector, multisubjects' focuses on creating the conditions of choice for a university's educational environment and providing each person with the opportunity for a personal move toward success, responsible decision-making, and developing themselves according to the demand of the different stakeholders of education and industry sector. More specifically, that choice consists of determining the individual path and pace of education, attaining a different level of education, and choosing the type of institutions, the subjects of education and teachers, the forms and methods of teaching, the means and methods of individual instruction, and the synergistic principles of pedagogy. The study results made it possible to determine key elements and trends in the organization, such as introducing a new framework as an “intervention-approach-synergy-integration” mechanism. This study advocates an adoption of a synergetic governance to accelerate the commercial quality of vocational education.

## Data availability statement

The original contributions presented in the study are included in the article/supplementary material, further inquiries can be directed to the corresponding author/s.

## Author contributions

MW and MS have designed the research plan, collected data, analyzed data, and written and revised the manuscript. Both authors have checked the final version of the manuscript and approved it for publication.

## Funding

This study is funded by National Social Science Fund of China Education Science Western Region Project entitled “The Behavior Logic and Realization Mechanism of Multiple Subjects' Synergetic Governance on Vocational Education” and grant number: XJA190284.

## Conflict of interest

The authors declare that the research was conducted in the absence of any commercial or financial relationships that could be construed as a potential conflict of interest.

## Publisher's note

All claims expressed in this article are solely those of the authors and do not necessarily represent those of their affiliated organizations, or those of the publisher, the editors and the reviewers. Any product that may be evaluated in this article, or claim that may be made by its manufacturer, is not guaranteed or endorsed by the publisher.
